# Genome-Wide Identification of Wheat ZIP Gene Family and Functional Characterization of the *TaZIP13-B* in Plants

**DOI:** 10.3389/fpls.2021.748146

**Published:** 2021-11-03

**Authors:** Song Li, Zihui Liu, Linlin Guo, Hongjie Li, Xiaojun Nie, Shoucheng Chai, Weijun Zheng

**Affiliations:** State Key Laboratory of Crop Stress Biology for Arid Areas, College of Agronomy, Northwest A&F University, Yangling, China

**Keywords:** wheat, ZIP gene family, expression profiles, yeast complementation, Zn/Fe stress, transgenic *Arabidopsis*, micro elements

## Abstract

The ZIP (Zn-regulated, iron-regulated transporter-like protein) transporter plays an important role in regulating the uptake, transport, and accumulation of microelements in plants. Although some studies have identified ZIP genes in wheat, the significance of this family is not well understood, particularly its involvement under Fe and Zn stresses. In this study, we comprehensively characterized the wheat ZIP family at the genomic level and performed functional verification of three TaZIP genes by yeast complementary analysis and of *TaZIP13-B* by transgenic *Arabidopsis*. Totally, 58 TaZIP genes were identified based on the genome-wide search against the latest wheat reference (IWGSC_V1.1). They were then classified into three groups, based on phylogenetic analysis, and the members within the same group shared the similar exon-intron structures and conserved motif compositions. Expression pattern analysis revealed that the most of TaZIP genes were highly expressed in the roots, and nine TaZIP genes displayed high expression at grain filling stage. When exposed to ZnSO_4_ and FeCl_3_ solutions, the TaZIP genes showed differential expression patterns. Additionally, six ZIP genes responded to zinc-iron deficiency. A total of 57 miRNA-TaZIP interactions were constructed based on the target relationship, and three miRNAs were downregulated when exposed to the ZnSO_4_ and FeCl_3_ stresses. Yeast complementation analysis proved that *TaZIP14-B*, *TaZIP13-B*, and *TaIRT2-A* could transport Zn and Fe. Finally, overexpression of *TaZIP13-B* in *Arabidopsis* showed that the transgenic plants displayed better tolerance to Fe/Zn stresses and could enrich more metallic elements in their seeds than wild-type *Arabidopsis*. This study systematically analyzed the genomic organization, gene structure, expression profiles, regulatory network, and the biological function of the ZIP family in wheat, providing better understanding of the regulatory roles of TaZIPs and contributing to improve nutrient quality in wheat crops.

## Introduction

Zinc (Zn) and iron (Fe), both essential in biochemical activities, are required for plant growth and development. Zn is an essential component for the metabolic enzymes that regulate enzymatic activity (Maret, 2004; Welch and Graham, 2004). Iron is also important for many enzymes, including cytochrome oxidase, peroxisome, and catalase, all of which play an important role in respiratory electron transport (Sadeghzadeh, 2013; Pinson et al., 2015). During photosynthesis, Zn is linked with carbohydrate inversion and is involved in chlorophyll synthesis. Fe is an essential for some chlorophyll protein complexes in chloroplasts (Palmgren et al., 2008). Although plant growth and development requires Zn and Fe, excessive amounts of Zn and Fe are harmful to the plant’s biological processes (Briat and Lebrun, 1999). As a result, plant cells have evolved multiform transport networks to balance the absorption, utilization, and storage of these metal trace elements (Kambe et al., 2004; Taylor et al., 2004). These systems include the ZIP (Zn-regulated, iron-regulated transporter-like protein), CDF (Cation-Diffusion Facilitator), and HMA (Heavy Metal ATPase) proteins (Colangelo and Guerinot, 2006; Ajeesh Krishna et al., 2020).

In plant, ZIP transporters are involved in transporting iron and metallic ions. Most ZIPs are composed of 326–425 amino acid residues. The number of transmembrane domains (TM) in ZIP transporters ranged from 7 to 8, while the TM length between III and IV varies and contains multiple histidine residues (Guerinot, 2000), that ZIP transporters combine metal ions to form octahedral, tetrahedral, and plane structures (Kavitha et al., 2015). The first ZIP gene was reported in *Arabidopsis*, and many ZIP genes have been identified in recent years (Eide et al., 1996; Guerinot, 2000; Pence et al., 2000; Tiong et al., 2015). The proteins of these genes can transport various divalent cations, including Fe^2+^, Zn^2+^, Mn^2+^, and Cd^2+^. Sixteen ZIP genes have been found in rice and *Arabidopsis* (Mäser et al., 2001; Chen et al., 2008).

The first ZIP gene identified was *AtIRT1* in *Arabidopsis*, which primarily transports iron and is expressed in the roots. *AtIRT1* was upregulated under iron-deficient conditions and is upregulated when exposed to a nickel solution (Eide et al., 1996). *AtIRT1* was proven to transport Fe and Zn by yeast complementation assays. Further research demonstrated the *irt1* mutant leaves were severely etiolate, with the leaf iron content decreased by 70% compared to the wild type (WT; Vert et al., 2002). As the *AtIRT1* transformed into *irt1* mutant, the etiolation phenotype was alleviated (Krämer et al., 2007). *AtIRT2* is another IRT gene in *Arabidopsis* that has a similar function to *AtIRT1*, it can restore the ability to transport iron in yeast mutants (Vert et al., 2001). *AtZIP1* and *AtZIP2* are two genes that primarily transport Zn. *AtZIP1* is primarily expressed in the root and leaf vein, while *AtZIP2* is highly expressed in the root column (Milner et al., 2013). Subcellular localization analysis revealed that the protein of *AtZIP1* is located on the vacuole membrane and the protein of *AtZIP2* is located on the plasma membrane. This difference in protein localization implies that *AtZIP1* and *AtZIP2* function keep differently. Functional validation revealed that *AtZIP1* plays a key role in the reactivation of metal ions transported from the vacuoles to the root cytoplasm, whereas *AtZIP2* is involved in Mn and Zn absorption from the roots. Both genes are crucial for a plant to absorb Mn and Zn through its root and transport them from the roots to the leaves (Milner et al., 2013). Previous studies have demonstrated that certain ZIP genes are involved in the response to Zn-deficiency in *Arabidopsis* (Grotz and Guerinot, 2006; Lee et al., 2010b).

*OsIRT1* and *OsIRT2* are the primary transporters of Fe in rice (Ishimaru et al., 2006; Li et al., 2019a). These two genes are mainly expressed in the roots and are significantly upregulated when rice is exposed to Fe-deficient conditions (Ishimaru et al., 2006; Itai et al., 2013). Overexpression of *OsIRT1* in rice causes rice sensitivity to Zn and Cd, while also increase resistance to Fe-deficient stress (Ishimaru et al., 2007; Nakanishi et al., 2010). At the seedling stage, there is no significant difference between the phenotype of the overexpression and the wild-type varieties; however, at the adult stage, the overexpression variety had shorter and fewer tillers and lower yields compared to the wild type, while the Fe and Zn contents in the grains increased (Lee and An, 2009; Lee et al., 2010b). *OsIRT2* was similar to *OsIRT1* in that the transport capability of Mn is less than *OsIRT1* (Nakanishi et al., 2010). Previous studies have revealed that the family members *OsZIP3*, *OsZIP4*, *OsZIP5*, and *OsZIP8* also transported Zn in rice (Ramesh et al., 2003; Lee and An, 2009; Lee et al., 2010a,b; Kavitha et al., 2015; Sasaki et al., 2015). Nine ZIP genes were identified in maize and were located on the plasma membrane and endomembrane system. Yeast complementation demonstrated that all ZmZIP proteins can restore the iron transporter mutant *fet3fet4*, and that *ZmIRT1* showed the strongest propagation under both Zn- and Fe-limited conditions (Li et al., 2013).

ZIP proteins have been widely investigated in model plants such as rice, maize, and *Arabidopsis* (Eide et al., 1996; Vert et al., 2001, 2002; Ishimaru et al., 2006; Li et al., 2013). However, few ZIP genes have been reported in wheat plants.

Sixteen ZIP genes have been identified in the wheat genome, though few studies have performed the expression analysis on these genomes (Tiong et al., 2015; Evens et al., 2017). Five ZIP genes in wheat were demonstrated to transport zinc and iron, these five ZIP genes were selected for analysis using yeast complementation since their sequence was similar to Zn-transporting ZIPs from *Arabidopsis*, rice, and barley (Tiong et al., 2014, 2015; Evens et al., 2017).

Wheat is a worldwide staple crop, feeding approximately 35% of the world’s population (Peng et al., 2011). As breeding technology and cultivation programs have increased, the production of wheat has risen. However, its nutritional quality has not improved: the Fe and Zn contents in wheat cannot meet human needs. Approximately two billion people suffer from Zn and Fe deficiency in South Asia and Sub-Saharan Africa. This deficiency has been called “hidden hunger,” and makes induces weight loss, cognitive impairment, anti-spasmodic decline, and often occurs in pregnant women, infants, and adolescents (Morgounov et al., 2007). Genetic engineering is the most convenient, effective, and durable method of increasing the Zn and Fe content in wheat grain. Therefore, it is critical to identify the genes involved in the uptake, transport, and enrichment of Zn and Fe in wheat. In this study, the ZIP genes in wheat were analyzed at the genomic level, three TaZIP genes were functionally validated by yeast complementation, and *TaZIP13-B* function was further verified by transgenic *Arabidopsis*. This research aims to uncover new candidate genes to improve the nutritional quality of wheat.

## Materials and Methods

### Plant Materials

Two wheat varieties (ZhongMai175 and Xiaobaimai) and one rice variety (*Oryza sativa* L. *japonica*. cv. Nipponbare) were utilized in this research. ZhongMai175 is a high Zn wheat variety that allowing for analysis of the expression of TaZIP genes in wheat (He et al., 2015). This line was planted at the experimental station of Northwest A&F University, Yangling, China (34°20'N, 108°24'E). Each row was 1m wide with four duplicates, while the row spacing was 0.25m and the plant spacing in each row was 0.05m. At the 7days after flowering stage (7 DAF), we collected grain sample from one plant in each row which was mixed with liquid nitrogen. One week later, the second sample was obtained (14 DAF). Four samples were collected in this study; there are 7, 14, 21, and 28 DAF.

Xiaobaimai is a landrace, drought-tolerant wheat variety found in PingYao city (ShanXi Province, China). This variety contained low zinc and iron in grain. Culturing in a glass petri dish with two layers of filter paper containing 1/4 Hoagland solution and place at climate chambers (RXZ-500D-LED, Ning Bo) at a light/dark cycle of 16/8h at 24°C for 10days (Seedling two leaf). Then for analyzed the expression under Fe and Zn stress, wheat treated with Hoagland medium containing different concentrations of ZnSO_4_ and FeCl_3_ solutions for 1h. In this study, the concentration of ZnSO_4_ and FeCl_3_ solutions was 0.05, 0.5, 50μmol/L. For investigated the expression under Zn- and Fe- deficient conditions, we treated it with Hoagland medium lacking ZnSO_4_ (Zn-deficient), Fe (III)-EDTA (Fe-deficient).

### Identification and Bioinformatics Analyses of TaZIP Genes

The sequence of the wheat proteins was downloaded from the Ensembl Plant database,^1^ after which the HMM profile for the ZIP DNA-binding domain (PF02535) was downloaded from the Pfam v31.0 database^2^ to search against the plant protein sequences using a threshold of *E*<1e^−5^ (Finn et al., 2016). Blast and manual corrections were then performed to remove alternative events and redundancy. The OsZIP genes were downloaded from the NCBI database,^3^ according to the methods used by Chen and Tiong (Chen et al., 2008; Tiong et al., 2014). The NJ phylogenetic tree was constructed with MEGA 7 and EvolView^4^ based on the wheat and rice protein sequences, with 1,000 bootstrap replicates. Putative TMHs of TaZIPs were predicted using the TMHMM Server v.2.0 (Krogh et al., 2001). Subcellular location was predicted by the WoLF PSORT.^5^

### Gene Structure and Conserved Motif Analyses

Gene structure was analyzed by GSDS.^6^ Protein conserved motifs were predicted using the MEME Suite web server,^7^ with the number of motifs set to 10, at a width range from 5 to 200 amino acids.

### TaZIP Genes and miRNA Co-expression Networks Construction

The miRNA target to the TaZIP genes was searched using the psRNATarget tool (Dai and Zhao, 2011), and the TaZIP cascade transcript was submitted in the miRBase. The cytoscape tool^8^ was used to visualize the regulatory network of the-miRNA and TaZIP genes.

### RNA and miRNA Isolate and Expression Pattern Analysis

For wheat tissue expression pattern analysis, the expression pattern data were downloaded from the RNA-seq database.^9^ For the expression pattern at grain filling stage and under Zn and Fe stress assay, RT-qPCR was used. Total RNA was isolated from the wheat grains and wheat, rice leaves using an RNAprep Pure Plant Kit (Tiangen, Beijing, China) and from the seedlings with TRIZOL (Takara, Dalian, China). cDNA synthesis was performed in a 20μl reaction mixture containing 1μg of total RNA and a mixture of TIANscript RT Kit (Tiangen, Beijing, China). The real-time PCR mixture contained 1μl cDNA, 1μl forward and reverse primers, and 17μl SYBR Green (Tiangen, Beijing, China). Real-time qPCR was performed in an ABI7300 (Thermo Fisher Scientific, United States) Real-TimeThermal Cycler and repeated three times. The actin of the wheat genes (Gene ID: AB181991) was used as a control. The 2^−∆∆*C*t^ method was used for fluorescence quantitative data analysis (Livak and Schmittgen, 2001).

The miRNAs of the wheat seedling under ZnSO_4_ and FeCl_3_ stresses were extracted using a miRcute miRNA Isolation Kit (Tiangen KR211, Beijing, China). miRNA-cDNA synthesis was performed with miRcute and miRNA First-Strand cDNA Kit (Tiangen KR211, Beijing, China). The real-time reaction mixture was performed with miRcute Plus miRNA qPCR Kit (SYBR Green; Tiangen FP411, Beijing, China) with three biological replicates. The qPCR reaction conditions were 95°C for 15min, followed by 45cycles of 94°C for 20s, and 58–60°C for 34s. Data of miRNA-qPCR were analyzed using the 2^−∆∆*C*t^ method.

### Cloning of TaZIP and OsZIP Genes

The CDS and ORF sequences were obtained from the Wheat Sequence database.^10^ The primers were designed with Oligo 7, with three genes cloned. In this step, the RNA of Xiaobaimai and *Oryza sativa* L. seedling was used for cDNA synthesis. The PCR reaction solution was 50μl and contained the following: 5μl cDNA as an amplification templet, 2.5μl forward and reverse primers, 25μl 2×master Mix, and 15μl Nuclease free water (NEB, United States). The reaction solution was performed on a DNA amplification machine (Thermo Fisher Scientific, United States). PCR amplification procedures were as follows: initial denaturation at 98°C for 30s, followed by 35cycles of denaturation at 98°C for 10s, annealing at 60°C for 20s, extension at 72°C for 30s, while the final extension was at 72°C for 2min. After amplification, we added 7μl purple 2-Log Ladder (NEB, United States) to the PCR products and separated them on 1.5% agarose gel for 30min at 120V. After they were separated, the PCR products were purified using a Universal DNA Purification Kit (Tiangen, Beijing, China) and connected to the cloning vector pLB (Tiangen, Beijing, China), and sequenced.

### Yeast Complementation

Specific primers were designed for PCR amplification and constructing the expression vector. The PCR procedure is the same as above, except for the annealing, which took place at 70°C for 20s. The PCR products were spread on agarose gel and linked to the *BamH I* site of the yeast expression vector pDR195 (PLASMID, China). They were subsequently sequenced and transformed into yeast competent cells. The yeast competent cells were prepared according to the methods used by Gietz and Schiestl (1995). Three yeast strains were used in this experiment: DY1455 (MATa ade6 can1 his3 leu2 trp1 ura3), *fet3fet4* DEY1453 (MATa/MATa ade2/+ can1/can1 his3/his3 leu2/leu2 trp1/trp1 ura3/ura3 fet3-2::HIS3/fet3-2::HIS3 fet4-1::LEU2/fet4-1::LEU2), and *zrt1zrt2* ZHY3 (MATa ade6 can1 his3 leu2 trp1 ura3 zrt1::LEU2 zrt2::HIS3; Li et al., 2013). The pDR195-TaZIPs were converted to DEY1453 and ZHY3 with the lithium acetate conversion method used by Gietz and Schiestl (1995). To verity the experiment was performed properly, *OsIRT1*, *OsZIP3*, and *OsZIP5* as well as converted to yeast competent cells as positive controls, the wild-type strain DY1455 harboring pDR195 was also used as a positive control. The empty vector pDR195 was used as a negative control converted to two yeast mutants. Transformed cells were coated on the selective SD-URA solid medium without corresponding amino acids. To verify the gene function, we diluted the yeast liquid OD_600_ to 1, 0.1, 0.01, 0.001, and dropped 10μl yeast liquid onto a different medium. The yeast strain of *zrt1zrt2* ZHY3 was grown on an SD/–ura medium (pH 4.4) supplemented with 0.4mM EDTA or 300μM ZnSO_4_. The yeast strain of *fet3fet4* DEY1453 was grown on SD/–ura medium (pH 5.8) containing 50mM 2-(4-Morpholino) ethanesulfonic acid (MES) supplemented or 200μM FeCl_3_.

### Phonotype Analysis in *Arabidopsis*

Specific primers were used for vector construction, the expression vector PCAMBIA1302 and the *Nco I* site were used for gene construction. After sequencing, the overexpression plasmid of PCAMBIA1302-TaZIP13 was transferred into the GV3101 (*Agrobacterium tumefaciens*) strain and transformed into *Arabidopsis*. The transgenic lines were cultured in a light temperature incubator until they were propagated for the third generation. The homozygous plants of the T3 progeny and WT were used for further study.

For the germination assays, the seeds of wild type and transgenic lines were surface sterilized and kept at 4°C for 72h in the dark before germination. About 25 seeds of every genotype were sown on the same plate containing different concentration of FeCl_3_ and ZnSO_4_ solution MS medium at 22/20°C (day/night) with a photoperiod of 16/8h (day/night) for 7days. Each day germinated seeds with protruded radicles were counted. The concentration of FeCl_3_ and ZnSO_4_ solution was 0, 50, 200, 300μmol/L. After germinated seeding were counted, four *Arabidopsis* lines were cultured until they were at six leaf stage, then the root length of 30 seedings from each line was measured and photographed. On the other hand, four lines from MS medium were transplanted into soil and treated with different concentrations of FeCl_3_ and ZnSO_4_ to analyze the tolerance. In this study, the concentration of ZnSO_4_ and FeCl_3_ was 200, 300, 400μmol/L. After 2weeks, a 0.15g of each sample leaf was collected and determine the chlorophyll content according to the methods used by Richardson et al. (2010). And three leaves of each line were collected and taken the midsection epidermis of the leaves to observe the stomas.

### Seeds of Light Microscopy, and Root Length, Fe and Zn Contents

Four lines were treated with water until maturity. Then seeds from the siliques located in the basis of a major inflorescence were selected for observation. Seeds from WT and transgenic lines were randomly selected, then photographed using stereomicroscope (Olympus mzx7) and a test instrument (WAN SHENG, HiCC-A). For the Zn and Fe content assays, 0.15g plant shoots, roots, and seeds of *Arabidopsis* were collected and digested in 2ml HNO_3_ overnight, then 2ml H_2_O_2_ was added and completed the digestion by microwave treatment, after which the digests were diluted with Millipore-purified water and filtered. The volume was then adjusted to 25ml, was ICP-OES analyses using an ICAP 6000 Series spectrometer (Thermo-Fisher; Hansen et al., 2013). For metal content measurements, three samples were conducted for each line. Each sample (shoots, roots, and seeds, respectively) was a mix of 15 plants.

### Statistical Analysis

The length of roots, length and width of seeds were counted by ImageJ software (Rueden et al., 2017). Data were analyzed and graphs were drawn using Excel 2019 (Microsoft Corporation, United States). In all graphs, error bars indicate standard deviation, and significant differences are indicated with ^*^p<0.05 or ^**^p<0.01.

## Results

### Identification and Classification of ZIP Genes in Wheat

**Figure 1:**
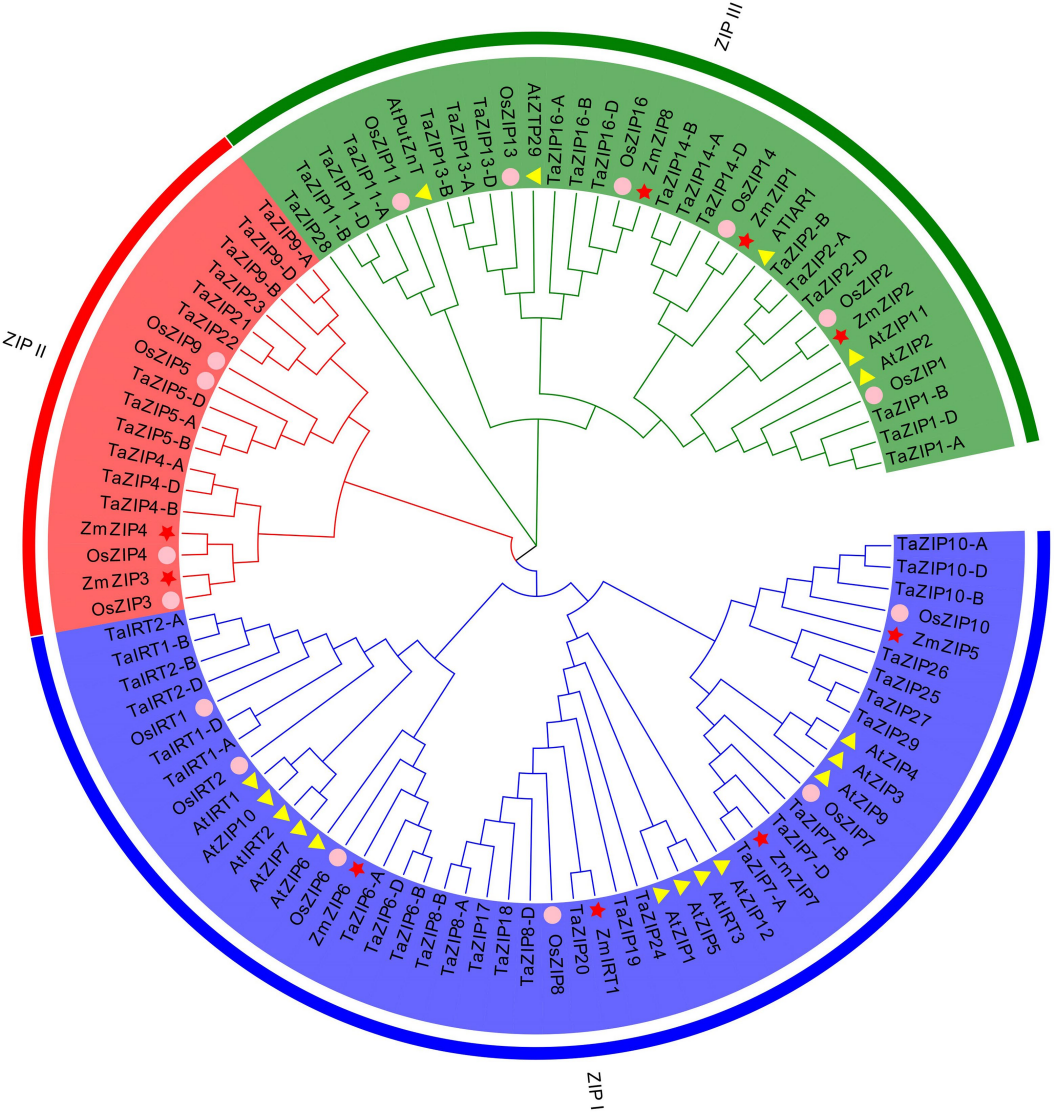
Phylogenetic tree of ZIP (Zn-regulated, iron-regulated transporter-like protein) proteins based on the full-length protein sequences using the neighbor-joining method. The three different groups are indicated by different colors. The proteins of rice, *Arabidopsis*, and maize are indicated by different shapes.

A total of 58 ZIP genes were identified using a whole-genome search (Figure 1; Supplementary Table S1; Supplementary Figures S1, S2), of which 44 wheat ZIP genes were found to share an orthologous relationship with rice. These were named according to rice-related nomenclature (Figure 1). The remaining genes were named from *TaZIP17* to *TaZIP30*, based on their location on the chromosome, from 1A to 7D (Supplementary Figure S2). Our results demonstrated that the TaZIPs were unevenly distributed on the chromosomes, and no ZIP genes located on the fifth chromosome group. The length of the TaZIP amino acids ranged from 185 to 577 and contained between 3 and 13 transmembrane domains. Most contained between 7 and 9 TM, while the length between TM-3 and TM-4 varied. Subcellular localization of the TaZIP genes was found to be on the plasma membrane and nucleus (Supplementary Table S1).

We constructed the phylogenetic relationship of the wheat ZIPs with rice, maize, and *Arabidopsis* ZIP proteins (Figure 1 and Supplementary Tables S1 and S2). The results revealed that these ZIP proteins were classified into three groups. Group ZIPI and group ZIPIII included all species proteins, suggesting that these ZIP proteins have a conserved function in monocots and dicots. Only three monocot species were included in group ZIPII, including *Arabidopsis*, suggesting that these proteins are unique to monocot. The wheat proteins are consistent with those of rice and maize, except for *ZmZIP5* and *ZmZIP1*. Six wheat ZIP genes showed a close relationship with *OsIRT1*, *OsIRT2*, *AtIRT1*, and *AtIRT2*, suggesting that these six TaZIPs shared a similar function with *OsIRT1*, *OsIRT2*, *AtIRT1*, and *AtIRT2*, all of which are involved in Fe transport in wheat. The wheat proteins are evenly distributed on three branches.

### Gene Structure and Conserved Motifs of TaZIP Genes

**Figure 2:**
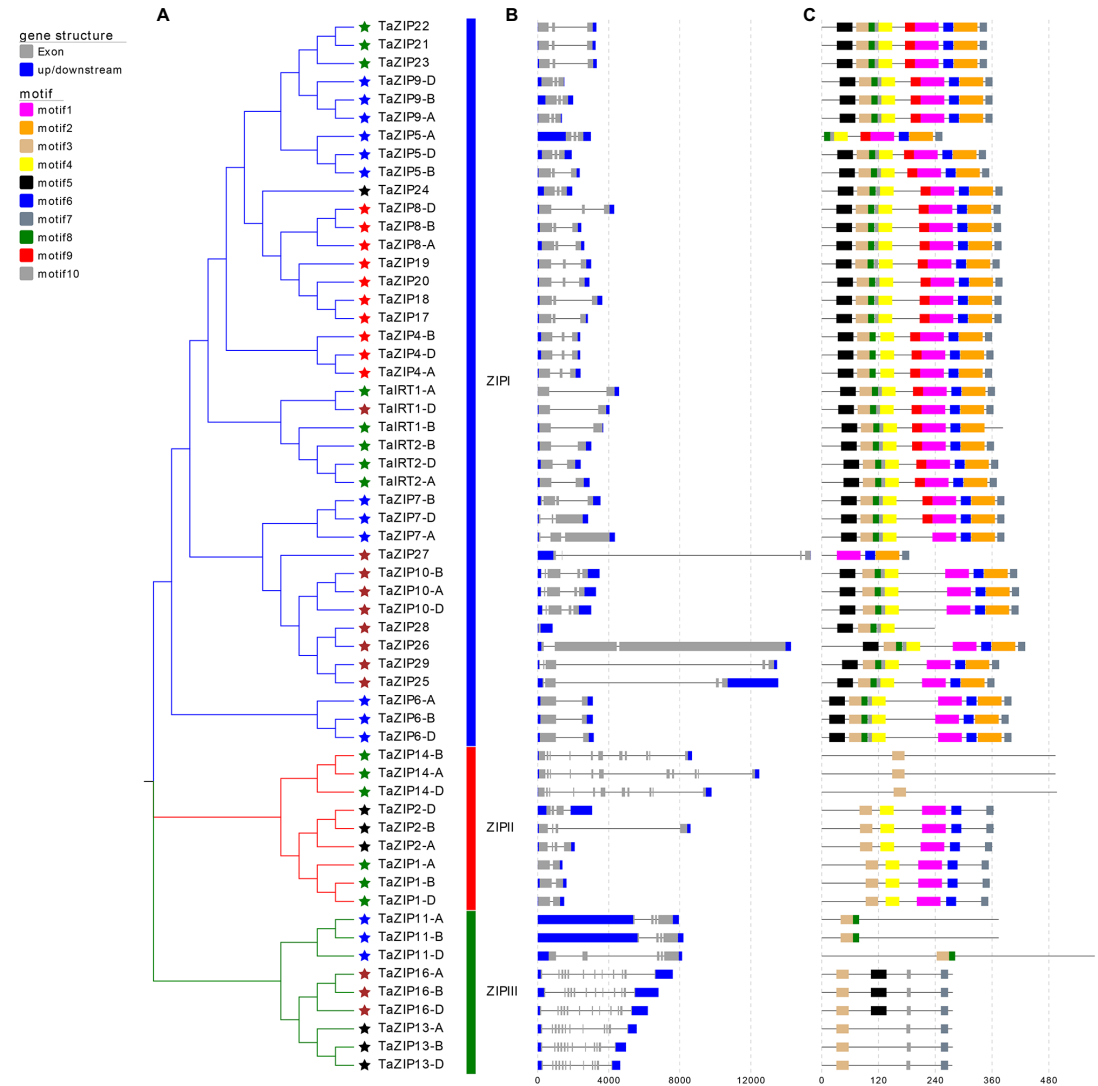
Gene structures and conserved motifs of these identified 58 TaZIP proteins. **(A)** Phylogenetic relationship of these 58 TaZIP genes; **(B)** exon-intron structures of these TaZIP genes. Blue boxes represent UTRs, gray boxes represent exons, and gray lines represent introns; **(C)** conserved protein motifs of these TaZIP proteins. The boxes in different colors represent different motifs, and the gray lines represent non-conserved sequences.

To comprehensively understand the function of TaZIPs, we analyzed their gene structure and conserved motifs (Figure 2). ZIP size ranged from 836 to 14,494bp (Supplementary Table S1). Of these, the *TaZIP28* gene was the shortest and the *TaZIP27* gene was the longest. The number of introns varied from 0 to 11, and the number of exons ranged from 1 to 12. *TaZIP27* had the largest size, *TaZIP16-A, B, D* and *TaZIP13-A, B, D* had the most exons (up to 12). In addition, *TaZIP28* has only one exon. Genes sharing a closer phylogenetic relationship had a more similar gene structure.

Using the MEME tool, 10 conserved motifs were identified in the wheat ZIPs (Figure 2 and Supplementary Table S3). The conserved motifs of the same group were similarly organized. Almost all TaZIP proteins possess motif 3 because it contains a histidine residue that binds to metal ions for transmembrane transport. All TaZIP proteins in group ZIPI had motif 1 through motif 7, except for four truncated genes that lacked some of these motifs (*TaZIP5-A*, *TaIRT1-D*, *TaZIP27*, and *TaZIP28*). In group ZIPII, three TaZIP proteins (*TaZIP14-A, B, D*) only had motif 3, while other members have the same motif. In group ZIPIII, motif 8 was detected in *TaZIP11-A, B, D*, and motif 5 was only detected in *TaZIP16-A, B, D*. Group ZIPI had the most motifs, while group ZIPII and ZIPIII had different quantities of motifs.

### Network Construction of TaZIP Cascade Genes

**Figure 3:**
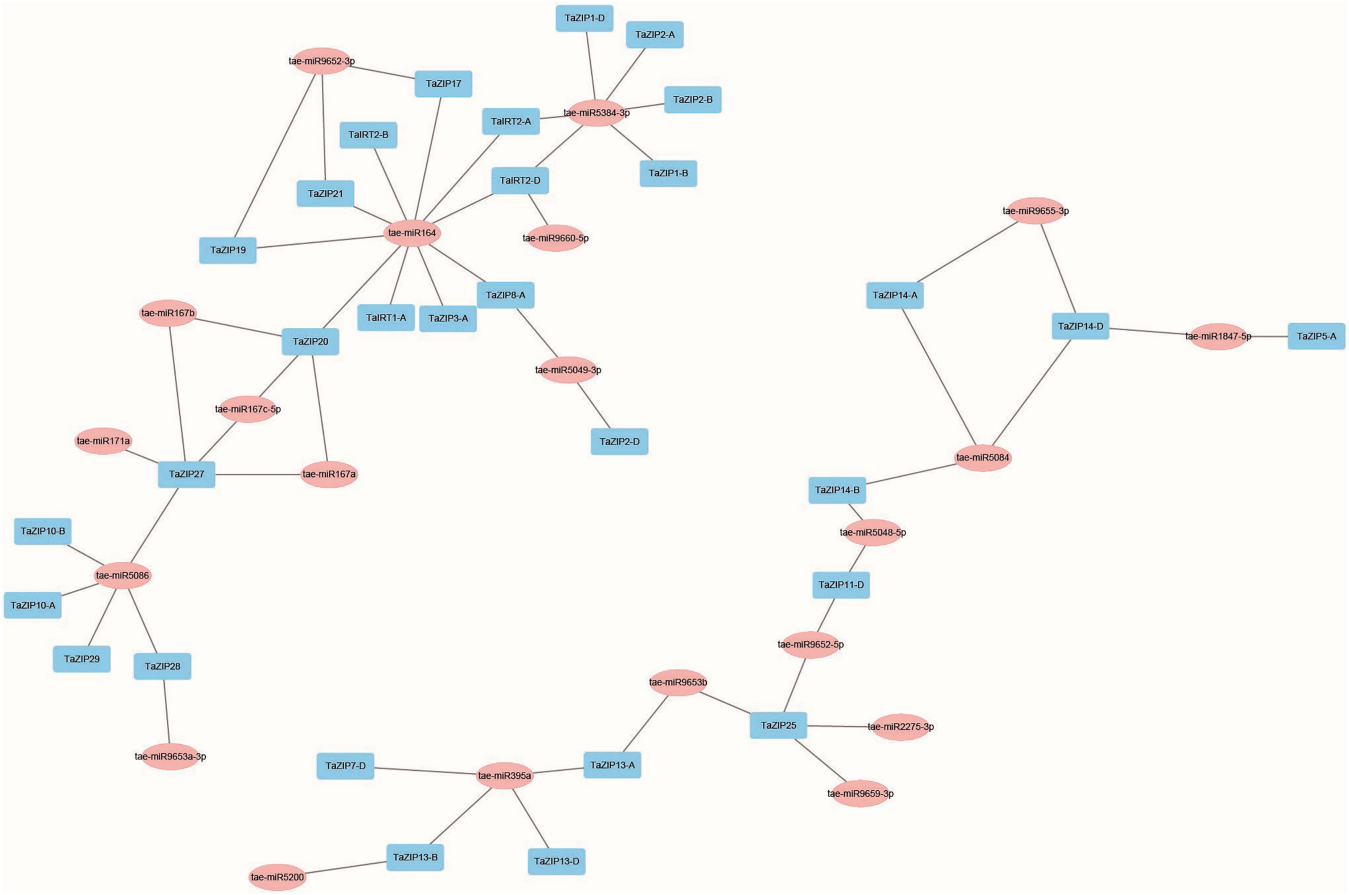
Co-expression network of TaZIP cascade genes in wheat. Blue box: TaZIP gene in wheat; pink box: miRNAs found in wheat.

The putative miRNA-targeted TaZIP genes were analyzed to assess the network of miRNA and TaZIP genes. Our results demonstrated that 20 miRNAs were predicted to target 30 TaZIP genes, while 28 TaZIP genes were not targeted by miRNA. This could be due to the current limitations on wheat miRNA (Supplementary Table S4). Based on the target relationship, 57 miRNA-TaZIP interactions were constructed (Figure 3). The wheat ZIP genes were inhibited by miRNA *via* translation (57.89%), while the rest of the genes were inhibited *via* cleavage (42.11%). Additionally, the miRNAs are primarily targeted in the CDS region but ahead of the ZIP domain of the TaZIP genes, silencing gene expression.

Furthermore, we constructed the co-expression regulatory network to detect the interaction between the TaZIP genes and miRNAs using a dataset of 173 RNA-seq, based on the weighted correlation of their expression.^11^ tae-miR164 and tae-miR5384-3p had major target genes, 10 and 6, respectively, while the *TaZIP25* and *TaZIP27* genes were targeted by the greatest number of tae-miRNAs (five tae-miRNAs; Figure 3 and Supplementary Table S4). Other genes targeted by a major number of tae-miRNAs were *TaZIP20* (targeted by four tae-miRNAs), *TaIRT2-D, TaZIP13-A*, and *TaZIP14-D* (targeted by three tae-miRNAs). Three genes of *TaIRT2-A*, *TaZIP14-B*, and *TaZIP13-B* were targeted by two tae-miRNAs. Figure 3 demonstrates that miRNA164 targeted *TaIRT2-A*, miRNA9660-5p targeted *TaZIP13-A*, and tae-miR5084 targeted *TaZIP14-B*.

### Expression Patterns of TaZIP Genes in Four Tissue and Grain Filling Stage

**Figure 4:**
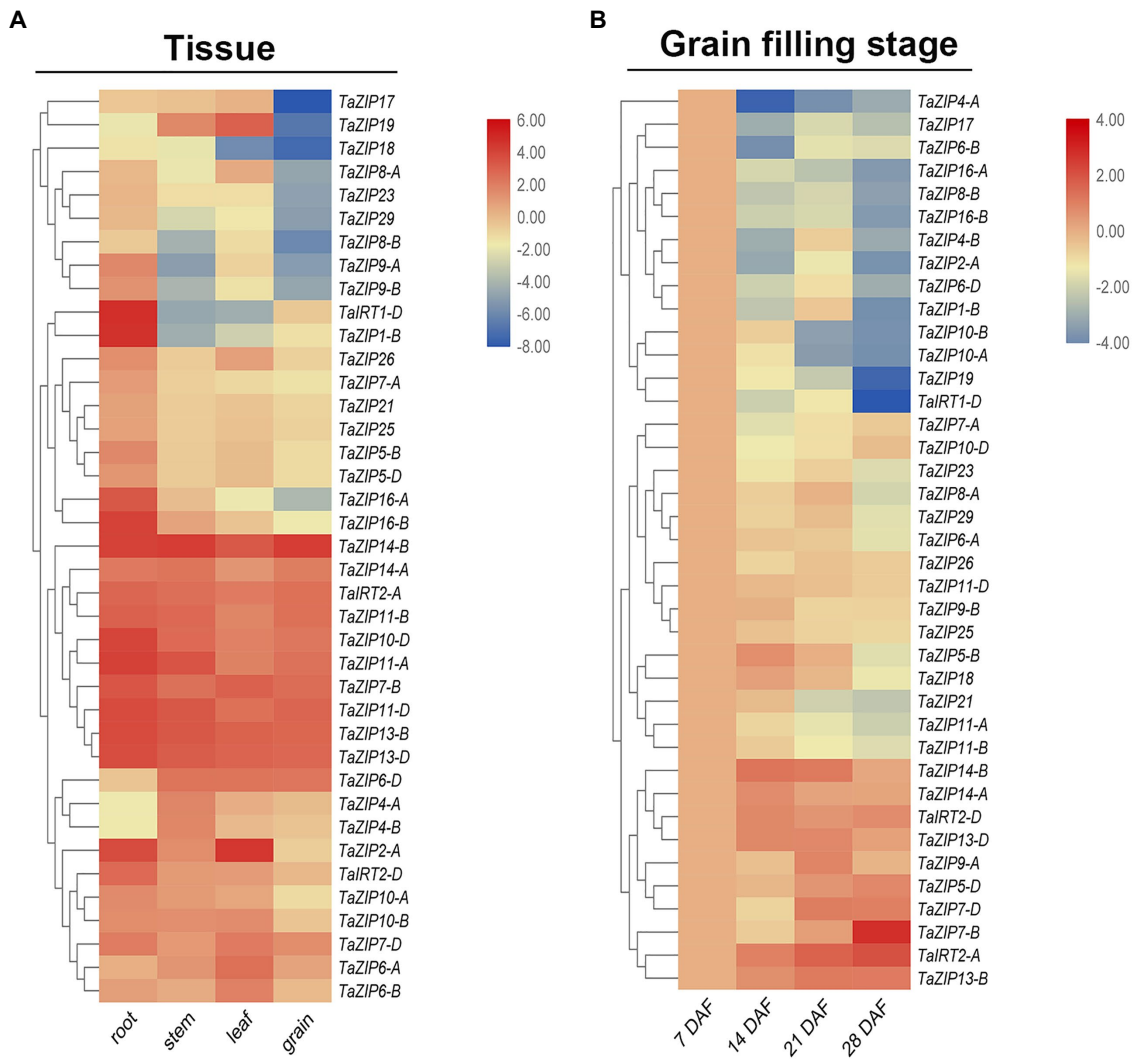
Expression profiles of TaZIP genes in different tissues **(A)** and at different grain filling stages **(B)**. **(A)** RNA-seq data of roots, leaves, stems, and grains of the genotype Chinese Spring were download for the URGI database and used for expression profiles analysis. The expression level was determined by the fragments per kilobase per million (FPKM) calculated by StringTie v2.1.2 tool and log2 transformation was used for normalization. **(B)** The grain of genotype ZhongMai175 at different grain filling stages were collected and then used for qRT-PCR. The expression level was calculated according to the 2^−∆∆*C*t^ method. Relative mRNA abundance of each gene was normalized with TaActin gene. DAF means day after flowering. The error bars indicate standard deviations. Three genes highlighted in yellow were used for cloned for downstream study.

Overexpression of some ZIP genes may enhance zinc and iron content, thus enhancing grain and fruit quality. RNA-Seq data were downloaded for analyzed the tissue expression pattern. To investigate the expression pattern of the TaZIP genes in wheat grain, we designed 39 primers for the fluorescence quantification of wheat ZIP genes (Supplementary Table S5). In total, 39 ZIP genes are expressed in wheat four tissues. Most were highly expressed in the roots but less in the grain (Figure 4A and Supplementary Table S6). Four genes (*TaZIP4-B*, *TaZIP4-A*, *TaZIP6-D*, and *TaZIP14-B*) were highly expressed in the stem and three genes (*TaZIP19*, *TaZIP6-A*, and *TaZIP2-A*) were highly expressed in the leaves. Except for *TaZIP16-A* and *TaZIP16-B*, group ZIPIII was highly expressed in four tissues, particularly in the grain (Figure 4). There were also differential expressions pattern between homoeologs genes: *TaIRT2-A* and *TaIRT2-B* displayed high expression in wheat grain, while *TaIRT1-D* displayed low expression in wheat grain (Figure 4A and Supplementary Table S6).

We utilized gene expression levels at seven DAF as a control to better understand the expression pattern of TaZIP genes during the grain filling stage. The results showed that TaZIPs have significantly different expression levels (Figure 4B and Supplementary Table S6). Thirty-one TaZIP genes displayed downregulated expression, with the lowest expression levels at 28 DAF. Eight TaZIP genes were highly expressed, but the expression trend was diverse, *TaZTP7-B* had highest expression in 28 DAF. At the grain filling stage, *TaIRT2-A*, *D* were also highly expressed. *TaZIP14-B* and *TaZIP14-A* were unique genes in that their expression was upregulated at the grain filling stage. The expression level of *TaZIP14-B* greatly increased, while *TaZIP14-A* was expressed moderately. Both *TaZIP13-B* and *TaZIP13-D* were highly expressed, but their expression patterns were different. The expression levels of *TaZIP13-D* increased rapidly while *TaZIP13-B* displayed moderate expression (Figure 4B and Supplementary Table S6).

### Expression Profiles of TaZIP Genes Under Zn and Fe Stress

**Figure 5:**
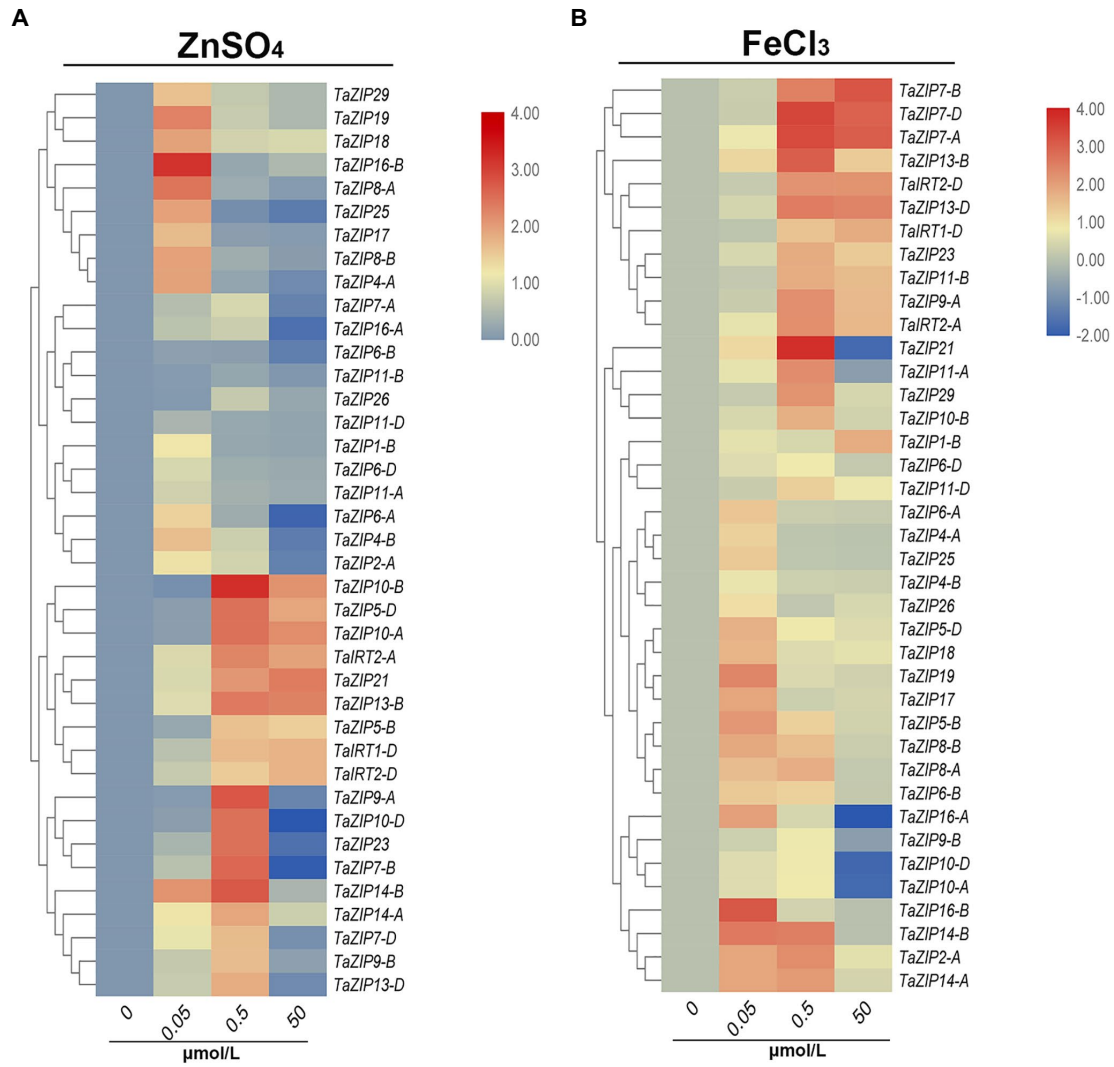
Expression profiles of TaZIP genes under Zn or Fe stress through qRT-PCR analysis. The wheat seedling at two leaf stage of genotype Xiaobaimai were treated under standard nutrient condition (CK), 0.05, 0.5, 50 ZnSO_4_ **(A)** and FeCl_3_ **(B)** and the samples were harvested at 1h after treatment. Data from qRT-PCR were analyzed according to the 2^−∆∆*C*t^ method. Relative mRNA abundance of each gene was normalized with TaActin gene. The error bars indicate standard deviations.

We then analyzed the expression patterns of ZIP genes under different concentrations of ZnSO_4_ and FeCl_3_ solutions. Under low concentrations of ZnSO_4_ stress, almost all ZIP genes were upregulated, but their expression trends differed. Sixteen ZIP genes were highly expressed (value more than 1) under 0.05μmol/L ZnSO_4_ and decreased under 0.5 and 50μmol/L conditions (Figure 5A). The expression of these genes was suppressed when Zn concentration increased. Eighteen genes were highly upregulated under the concentration of 0.50μmol/L ZnSO_4_. Moreover, the expression of nine ZIP genes (*TaZIP21*, -*TaZIP5-D*, *TaZIP5-B*, *TaZIP8-B*, *TaIRT1-D*, *TaIRT2-D*, *TaIRT2-A*, *TaZIP10-B*, and *TaZIP10-A*) increased in 50μmol/L ZnSO_4_. Especially *TaZIP14-B*, which displayed high levels of expression in the 0.5μmol/L solution, but low expressed in the 50μmol/L ZnSO_4_ solution. The *TaZIP13-B* gene was moderately expressed in the 0.05μmol/L solution, while it was upregulated in other concentrations of ZnSO_4_ solution (Figure 5A and Supplementary Table S6).

The expression pattern of TaZIPs in FeCl_3_ was similar to ZnSO_4_. At low concentrations of FeCl_3_ solution, the expression of all ZIP genes was increased, but of which 16 genes were further suppressed or moderately upregulated under 0.5 and 50μmol/L FeCl_3_ treatment (Figure 5B and Supplementary Table S6). Furthermore, nine genes were highly expressed in the 0.05 and 0.5μmol/L FeCl_3_ solutions. Eleven genes were highly expressed under 0.05 and 0.5μmol/L of FeCl_3_ treatment. The genes *TaZIP14-B*, *TaZIP13-B*, and *TaZIP7-A, B, D* were all upregulated under high concentrations of FeCl_3_ solution. The *TaIRT1-D* gene is key to transporting iron ions; however, its expression level under FeCl_3_ treatment is lower than under ZnSO_4_ treatment. Additionally, the expression pattern of *TaIRT2-A, D* was similar to that of *TaIRT1-D* (Figure 5B and Supplementary Table S6). We also performed Fe and Zn starvation treatment that wheat seedlings at two leaf stage were treated with Hoagland nutrient solution as CK and Hoagland medium lacking ZnSO_4_ (Zn-deficient) or Fe (III)-EDTA (Fe-deficient). After treated by 6 and 12h, six genes were upregulated in roots and shoots and mainly expressed in roots (Supplementary Figure S3). *TaIRT2-A, D* were upregulated slightly under Zn-deficiency while highly expressed under Fe-deficiency. *TaZIP13-B, D* and *TaZIP14-A, B* were highly expressed under Zn-deficiency condition.

### Expression Pattern of miRNA Under Zn and Fe Stress

To investigate whether miRNA degraded the ZIP genes, we analyzed the expression pattern of three miRNAs in wheat under ZnSO_4_ and FeCl_3_ solutions. These three miRNAs target *TaIRT2-A*, *TaZIP14-B*, and *TaZIP13-B* (Supplementary Tables S4 and S5).

**Figure 6:**
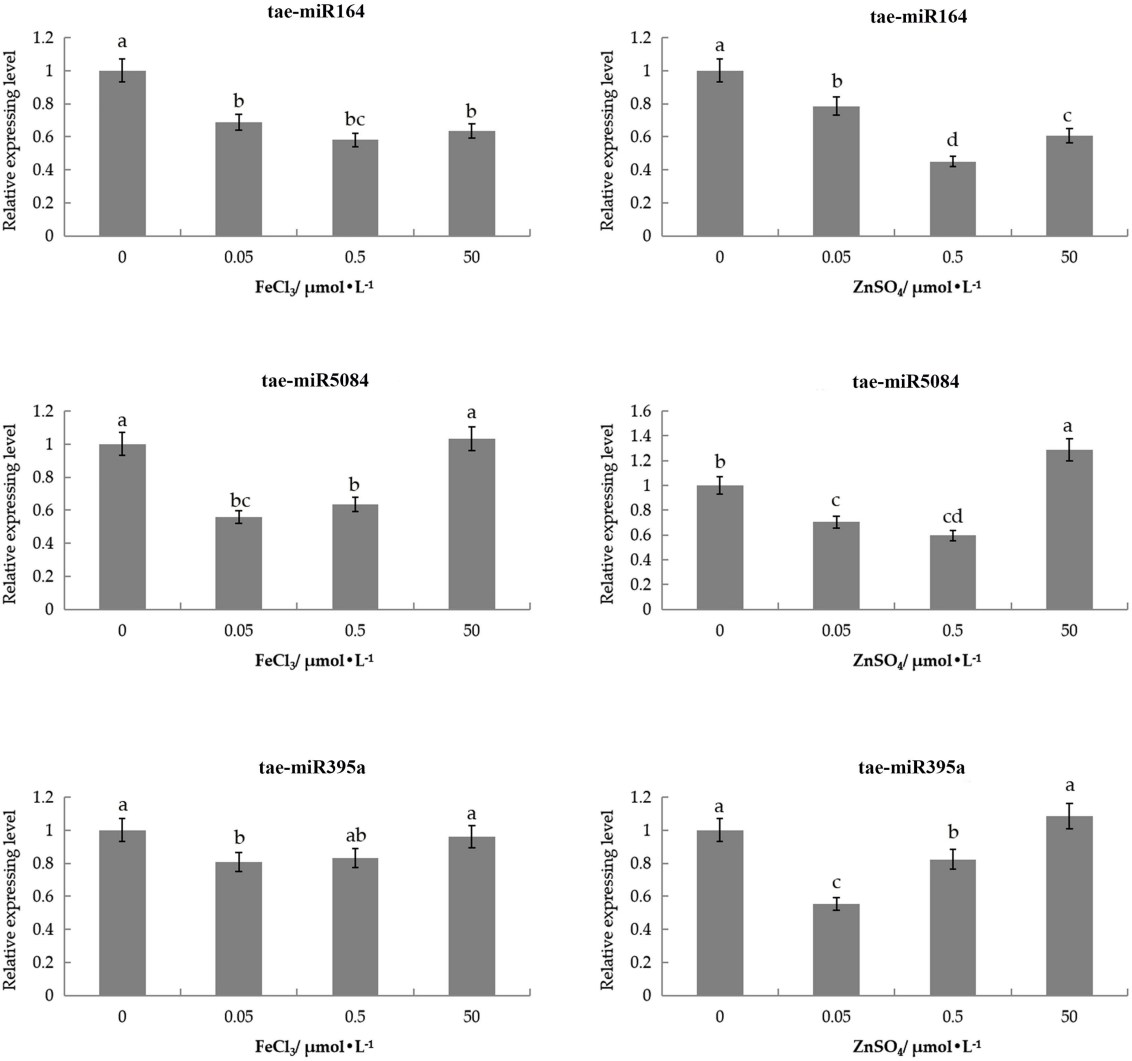
Expression patterns of three tae-miRNAs under Zn and Fe stress through qRT-PCR analysis. The expression level was calculated according to the 2^−∆∆*C*t^ method. Relative miRNA abundance of each gene was normalized with the expression of 0h. Error bars indicate the standard deviations, and different letters are significantly different.

Three miRNAs were slightly downregulated under 0.05 and 0.5μmol/L of the ZnSO_4_ solution compared with the control (Figure 6). tae-miR164 was also slightly downregulated in 50μmol/L ZnSO_4_ solution, while tae-miR5084miR5084 and tae-miR395a were slightly upregulated under 50μmol/L of the ZnSO_4_ solution.

tae-miR164 displayed low levels of expression in the FeCl_3_ solution compared with control, with the lowest expressed in 0.5μmol/L FeCl_3_ solutions. tae-miR395a and tae-miR5084 had similar expression patterns in the FeCl_3_ solution. These two miRNAs were downregulated in the 0.05 and 0.5μmol/L FeCl_3_ solutions, with the lowest expression in the 0.05μmol/l FeCl_3_ solution. However, under 50μmol/L of FeCl_3_, these two miRNAs were upregulated slightly and did not differ from the control (Figure 6). The expression pattern of miRNAs contrary to the targeted genes.

### Functional Analysis of Three TaZIPs by Complementation in Yeast Cells

After demonstrating that the three genes were upregulated when exposed to Zn and Fe stress, we also revealed the biological function of three ZIP genes (*TaZIP14-B*, *TaZIP13-B*, and *TaIRT2-A*) by yeast complementation analysis (Supplementary Table S5). *OsZIP3*, *OsZIP5*, and *OsIRT1* were chosen as positive controls, all of which have been demonstrated to be involved in Zn and Fe transport in rice (Chen et al., 2008; Tiong et al., 2014).

**Figure 7:**
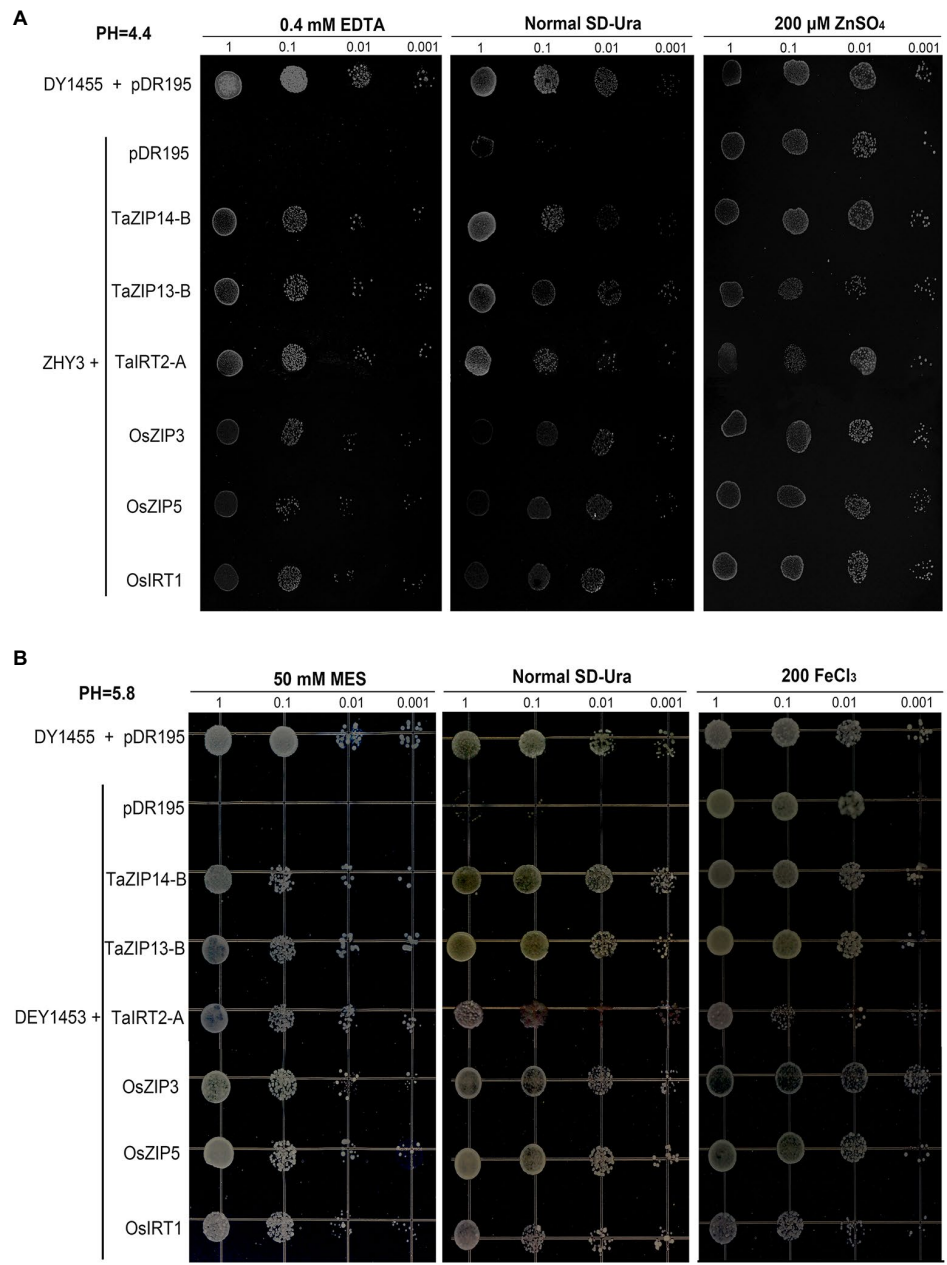
Functional complementation of yeast Zn and Fe transport mutants by TaZIPs under different pH conditions. **(A)** The Zn transport mutant *zrt1zrt2* (pH 4.4); **(B)** the Fe transport mutant *fet3fet4* (pH 5.5–5.8). Mutant transformed with the expression vector pDR195 carrying *TaIRT2-A, TaZIP14-B*, or *TaZIP13-B* or a functionally characterized ZIP gene, *OsZIP5, OsZIP8*, or *OsIRT1*. The wild-type (WT) strain DY1455 transformed with pDR195 was used as a positive control, and the yeast *zrt1zrt2* or fet3fet4 mutant transformed with the empty vector pDR195 was used as a negative control. The transformed yeast cells were grown under different metal conditions as indicated, and the transformed fet3fet4 was grown on medium with pH 5.8. Cell concentration was adjusted to OD_600_=1 and serial dilutions (1.0, 0.1, 0.01, and 0.001) were made. For assay, 5μl of each dilution was spotted on plates and grown for 6days at 30°C.

Three yeast strains wild-type DY1455, the Saccharomyces eviscerate *zrt1zrt2* mutant (ZHY3), and the *fet3fet4* mutant (DEY1453) were used, to verify that the three wheat TaZIP genes were capable of restoring the ability to transport zinc and iron in the mutant yeast. The full-length cDNA of both the wheat and rice genes were inserted and expressed in the two mutants. The transformed ZHY3 with TaZIP genes were grown on an SD medium with 0.4mM EDTA and the transformed DEY 1453 were grown on a SD medium with 50mM MES. The results demonstrated that the growth of the ZHY3 yeast with pDR195 was inhibited under zinc-deficient conditions in a normal SD-Ura medium, while the mutant with TaZIP genes and rice genes successfully recovered from the growth defect (Figure 7A). The *TaZIP13-B* gene reversed the growth defect. When the ZHY3 yeast was exposed to a 200μM ZnSO_4_ medium, the growth of ZHY3 was not inhibited. The growth of DEY1453 was similar to ZHY3 (Figure 7B). Under Fe-deficient conditions, the growth of DEY1453 containing a vector was severely inhibited, while the growth was reversed during the expression of TaZIP and rice genes. Once a sufficient amount of FeCl_3_ was supplied, growth recovered. *TaZIP13-B* demonstrated the strongest propagation under Fe-deficient conditions. These results revealed that *TaZIP14-B*, *TaZIP13-B*, and *TaIRT2-A* could effectively complement the zinc transporter mutant *zrt1zrt2* and the iron transporter mutant *fet3fet4*, suggesting they could successfully transport Zn and Fe.

### Gene Functional Analyzed by *Arabidopsis thaliana*

**Figure 8:**
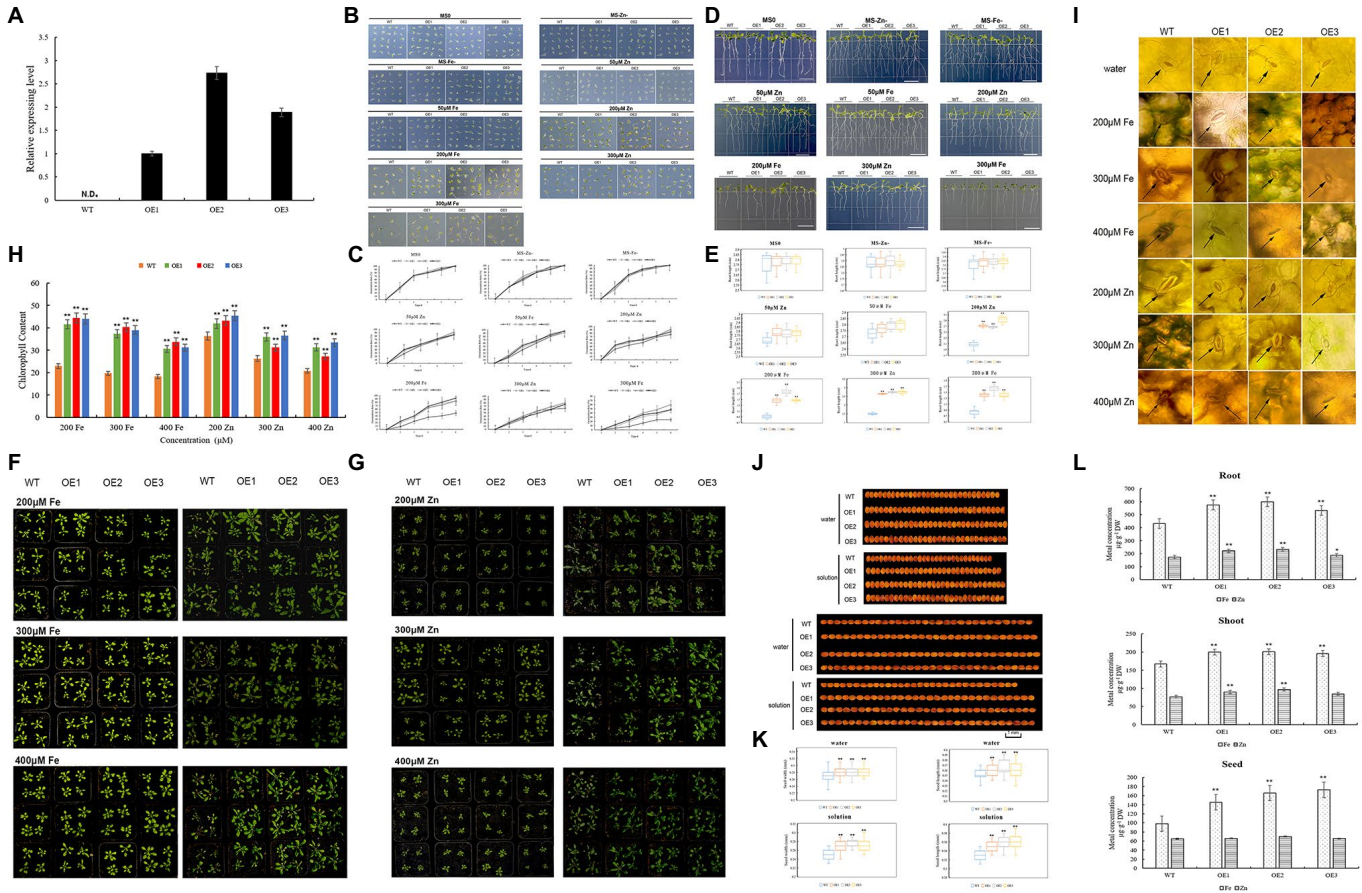
Comparison of the phenotypic performance of three *TaZIP13-B* overexpression *Arabidopsis* lines and wild type. **(A)** The expression level of *TaZIP13-B* in T3 transgenic *Arabidopsis* lines. Bars indicate standard deviations of three biological replicates; **(B,C)** seed germination rate with different treatment; **(D,E)** root length of WT and three transgenic lines (bar=1cm); **(F,G)** phenotypic identification of *Arabidopsis* treated with ZnSO_4_ and FeCl_3_ solution, respectively (concentration: 200, 300, 400μmol/L); **(H)** chlorophyll content; **(I)** phenotype of stomata; **(J,K)** seed width and length (bar=1mm, *n*>30); **(L)** the content of Zn and Fe in roots, shoots and seeds. Statistically significant differences are indicated: ^*^p<0.05; ^**^p<0.01 (Student’s *t*-test).

The *TaZIP13-B* gene was upregulated both at the grain filling stage and under Fe/Zn stress. The yeast complementation experiment proves that *TaZIP13-B* can transport Fe and Zn in a yeast mutant. Therefore, *TaZIP13-B* was transformed into *Arabidopsis* to verify this gene function. We selected lines OE-1, OE-2, and OE-3 with high expression levels for further analyses (Figure 8A).

On the MS, MS-Zn, and MS-Fe medium, the germination rate of the transgenic lines OE1, OE2, OE3, and WT approached 100%, with no significant difference between transgenic lines and WT (Figures 8B,C). When treated with 50μmol/L of Zn and Fe, the germination rate of four lines decreased, and no significant difference was observed between them (Figures 8B,C). When exposed to 200μmol/L of Zn and Fe MS medium, the germinations of the OE1-3 line were significantly higher than the WT line. The germination rate of OE1 was 85.3%, while that of OE2 was 82.7%, OE3 was 93.3%, and WT was 46.7% in 200μmol/L Fe MS medium. In 200μmol/L Zn MS, the germination rates of OE1-3 were 89.3, 90.7, and 85.3%, respectively, while that of WT was 74.7%. Compared with OE1 and OE3, the transgenic line OE2 had the highest germination on 300μmol/L Fe MS (up to 58.7%), while WT had a germination rate of only 22.7%. All three transgenic lines had significantly higher germination rates than WT line (Figures 8B,C).

Root length is another index used to evaluate plant tolerance to Zn and Fe stresses. We found no significant difference in root length between the three transgenic lines and WT on MS, Zn-, Fe-, and 50μmol/L Zn/Fe MS medium (Figures 8D,E); however, the number of roots in Zn-, Fe-, and 50μmol/L Zn/Fe MS medium increased. When transgenic lines and the WT line were placed on 200μmol/L Fe MS medium, the root lengths were significantly inhibited, but the root lengths of the three transgenic lines were significantly longer than WT line. The root length of OE2 was 1.78cm, which was the longest root length on the 200μmol/L Fe MS medium. On the 200μmol/L Zn MS medium, the three transgenic lines were significantly longer than the WT line. On the 300μmol/L Fe MS, the root lengths were further inhibited compared to the 200 Fe MS medium, and the root lengths of the three transgenic lines were significantly longer than the WT line. The root lengths were also inhibited in 300 MS Zn MS, though the root lengths of the three transgenic lines were significantly longer than that of the WT line (Figures 8D,E).

To further evaluate the tolerance of the three transgenic lines and WT to Zn and Fe stress, we treated the transgenic and WT lines with different concentrations of a ZnSO_4_ and FeCl_3_ solution. Under 200μmol/L FeCl_3_, the WT leaves wilted and yellowish-brown spots appeared on a few leaves, though this did not occur on the OE1, OE2, and OE3 lines (Figure 8F). Under 300 and 400μmol/L of the FeCl_3_ solution, most WT leaves were brown while transgenic lines were normal. Almost all of the WT petiole browned, particularly under 400μmol/L FeCl_3_ solution treatment (Figure 8E). We then measured the chlorophyll content of four lines. As the concentration of the FeCl_3_ solution increased, the chlorophyll content of the four lines decreased (Figures 8G,H). However, the chlorophyll content in the three transgenic lines was significantly higher than in the WT line. The three transgenic lines exhibit greater FeCl_3_ resistance than the WT line, according to observations of the stomata in all four lines (Figure 8I). The stomata opened on all four lines when treated with water. Under 200 FeCl_3_ μmol/L treatment, the stomata of the WT line closed slightly, while the stomata of the three overexpression lines opened. When treating the four lines with 300μmol/L FeCl_3_ solutions, the stomata of the WT closed while OE1, OE2, and OE3 remained open (Figure 8I). Under 400μmol/L FeCl_3_ solution, the stomata of all lines closed, and the stomata of the WT died. When treated with 200μmol/L ZnSO_4_ solution, the leaves of the WT line turned gray, while the leaves of the three transgenic lines remained green (Figure 8G). The results were similar to the 200μmol/L ZnSO_4_ treatment when they were treated with the 300 and 400μmol/L ZnSO_4_ solution. However, when treated with the 300 and 400μmol/L ZnSO_4_ solutions, part leaves in the transgenic lines turned gray and wilted (Figure 8G). The chlorophyll content of both of the transgenic and WT plants was analyzed. Under treatment with three different concentrations of ZnSO_4_ solution, the chlorophyll content in the WT line was significantly lower than in the transgenic lines (Figure 8H). The stomata phenotype of all lines under the ZnSO_4_ treatment was similar to that of the FeCl_3_ treatment (Figure 8I).

Zn is important to photosynthesis in plants, and photosynthesis is related to crop yield. Therefore, we analyzed whether the TaZIP genes affect seed size. When the four lines were treated with water, the seed width and length of the three transgenic lines were longer than that of the WT line (Figures 8I,J). When treated with 200mol/L FeCl_3_ and ZnSO_4_ solution, all lines’ seed size shrank, but the three transgenic lines’ seed breadth and length remained somewhat longer than the WT line (Figures 8J,K). Our results indicate that transferring the wheat *TaZIP13-B* gene into *Arabidopsis* increases seed size and might increase production.

We used 0.15g samples of the roots, shoots, and seeds to measure the metal contents of tissues in this study. Compared with the WT, the three overexpression lines accumulated more Fe (23.6–38% higher) and Zn (7.5–33% higher) in the roots, while overexpression lines also accumulated more Fe (17.3–20.3%) and Zn (10.3–26.0%) in the shoots than the WT line. Compared with the WT, the seeds of the transgenic lines have a higher Fe content and a higher Zn content (Figure 8L). These results indicate that transgenic lines can absorb more Fe and Zn from the soil, enriching the Fe and Zn in seeds.

## Discussion

Zinc and iron are two microelements that are essential for plant development. Inadequate zinc and iron can result in etiolation, wilting, and even death (Vert et al., 2001). The primary reason for zinc deficiency in plants is soil with low levels of Zn and Fe (Almendros et al., 2013). Approximately 30% of the world’s agricultural area is Zn-deficient, which affects both grain yield and the Zn concentration in grains (Paul, 2015). To achieve sustained Zn uptake from the environment, plants have a dual-transporter system that includes high- and low-affinity Zn transporters called ZIPs (transporter-like protein; Eide, 2006). This protein family has been reported in many species, including *Arabidopsis*, rice, barley, maize, and wheat (Bughio et al., 2002; Pedas et al., 2008; Li et al., 2013; Tiong et al., 2015; Evens et al., 2017). Previous studies have identified 42 ZIP genes in wheat, while many ZIP genes have not yet been identified (Tiong et al., 2015; Evens et al., 2017). In this study, we identified 58 ZIP genes in wheat, which includes 42 previously identified ZIP genes. Additionally, we analyzed the expression pattern both of specific tissues and under ZnSO_4_/FeCl_3_ treatment. We also analyzed the ZIP gene structure and the motifs of the TaZIPs. These genes were distributed on all chromosomes, except for chromosome 5. The localization of the ZIP genes was uneven, which could be due to the specific retention and dispersal of TaZIPs during polyploidization. The sequence length of wheat ZIP genes varied significantly, while the transmembrane domain between III and IV can be changed (Guerinot, 2000). The subcellular localization of the most TaZIPs proteins was predicted to be located on the membrane. Our results were consistent with those of ZmZIPs, AtZIPs, and HvZIPs (Lin et al., 2009; Li et al., 2013; Tiong et al., 2014). The plasma membrane is an important region for Zn and Fe transport since plant proteins located on the plasma membrane can quickly assimilate Zn and Fe from the environment (Schneider, 1983). Other ZIP proteins are located on the vacuolar membrane, including *AtZIP1* and *OsZIP6*. miRNA is a regulatory factor that plays an important role in regulating the expression level of plant proteins after transcription (Vaucheret, 2006). Therefore, we constructed the network of miRNAs and target genes and found that tae-miR164 and tae-miR5084 had the most targeted genes. These two miRNAs were targeted to TaIRT genes, in particular, tae-miR164 was targeted to *TaIRT1-A* and *TaIRT2-A, B, D*. This suggests that tae-miR164 and tae-miR5084 could each play an important role in the uptake and enrichment of Fe from the environment.

Most TaZIP genes are primarily expressed in the roots, while others are expressed in the leaves or stems. Our results demonstrate that the expression of most TaZIP genes in the roots helps absorb and transport Zn and Fe. Several studies have revealed that Zn and Fe were primarily absorbed by the roots and delivered to different tissues through the phloem-tropic mode (Yamaji and Ma, 2014). The Zn and Fe contents in grain is one of the most important indexes measuring wheat quality (Ziaeian and Malakouti, 2001). Zn and Fe accumulation typically occurs in the grain during the grain-filling stage (Tavarez et al., 2015). Some studies have demonstrated that mineral deficiency can induce the overexpression of ZIP genes (Li et al., 2013). Other studies have reported the relationship between the Zn and Fe content the overexpression of ZIP genes in cereal (Lee and An, 2009; Tiong et al., 2014). This study found that nine genes were upregulated at the grain-filling stage, indicating that these genes are likely involved in Zn and Fe accumulation in grain. *TaIRT2* expression levels were upregulated and *TaIRT1* expression levels were downregulated, which is similar to *OsIRT1* and *OsIRT2* (Nakanishi et al., 2010). In most plants, *IRT1* and *IRT2* have different transport substrates and different expression patterns during the plant growth stage (Vert et al., 2001; Pedas et al., 2008).

Most ZIP genes are upregulated under Zn- and Fe- deficient conditions (Mäser et al., 2001; Mizuno et al., 2008; Tiong et al., 2015; Evens et al., 2017). In *Arabidopsis*, *AtZIP1-5*, *AtZIP9-12*, and *AtIRT3* were induced by Zn-deficiency treatment; in rice, *OsIRT1* and *OsIRT2* were induced by Fe-deficiency treatment; and in wheat, *TaZIP3,-5,-7*, and *-13* were induced by Zn-deficiency treatment (Evens et al., 2017). The ZIP transporter is a dual-transporter system, which includes high-affinity and low-affinity Zn transporters (Sillanpaeae and AGL, 1982; Mizuno et al., 2008). The high-affinity system is saturated at approximately 0.1μmol/L, while the low-affinity system shows a linear relationship that varies from concentrations of 0.5 to 50μmol/L (Lee et al., 2010a,b). The Zn uptake system is a dual system in wheat (Reid et al., 1996). Our study demonstrated that approximately half of the TaZIP genes were highly expressed in the 0.05μmol/L ZnSO_4_ treatment, while others were highly expressed in the 0.5μmol/L ZnSO_4_ treatment. The TaZIP genes displayed a similar expression pattern in FeCl_3_ solution. Nineteen TaZIPs were highly expressed under 0.05μmol/L FeCl_3_ and 23 TaZIPs were highly expressed in 0.5μmol/L FeCl_3_. A previous study found that expression patterns of ZIP genes differed under different concentrations of Zn and Fe treatment. Based on Zn affinity, we considered the 16 TaZIP genes with the highest expression in the 0.05μmol/L ZnSO_4_ treatment to be high-affinity Zn transporters, while other TaZIP genes were considered low-affinity Zn transporters. In this study, we also found that four TaZIPs (*TaZIP18*, *TaZIP4-B*, *TaZIP29*, and *TaIRT2-A*) were upregulated under the 0.05 and 0.5μmol/L ZnSO_4_ treatments. We also prove that six ZIP genes were upregulated under Zn and Fe deficient conditions.

The regulatory role of miRNA inhibiting the expression of target genes, meaning that miRNAs and target genes have opposing expression patterns (Zamore et al., 2000; Bernstein et al., 2001). However, recent research has showed that miRNA also can activate gene transcription (Xiao et al., 2017). In this study, we analyzed the expression of three miRNAs under Zn and Fe stress. Our results demonstrated that three miRNAs could downregulated under low concentrations of ZnSO_4_ and FeCl_3_. However, tae-miR5084 and tae-miR395a were upregulated in 50μmol/L of ZnSO_4_ and FeCl_3_. Absorbing excessive Fe and Zn is toxic to plants, meaning that wheat may upregulate miRNA to inhibit TaZIP gene expression. The overexpression of tae-miR399-A1 could inhibit the expression of the *TaPHO2-A1, B1, D1* genes in a high-phosphorus aqueous solution, but wheat accumulates more Pi in its leaves (Ouyang et al., 2016). In this study, tae-miR5084 and tae-miR395a were both upregulated in 50μmol/L ZnSO_4_ and FeCl_3_, inhibiting the expression of targeted genes.

In yeast, the high-affinity transporter gene (*Zrt1*) is responsible for the uptake of Zn in a Zn-deficient medium. When Zn is abundant, *Zrt1* is repressed and the low-affinity transporter (*Zrt2*) mediates Zn uptake (Eide, 2006). ZHY3 is a yeast mutant that lack the *zrt1* and *zrt2* genes and unable grow on SD media without ZnSO_4_. *fet3fet4* DEY1453 is another mutant that cannot normally grow on the SD media without FeCl_3_. This growth deficiency may be reversed by inserting a functioning gene into these mutants. Yeast complementation has been used to demonstrate that ZIP genes can reverse growth defects in the *zrt1zrt2* and *fet3fet4* double mutant (Mäser et al., 2001). In this study, *TaZIP14-B*, *TaIRT2-A*, and *TaZIP13-B* inserted into the yeast mutant, and yeast complementation assays demonstrated that these genes could reverse the growth defect. Our results demonstrated that these three genes could effectively transport Zn and Fe. While some wheat ZIP genes have been studied, none of these three genes have been tested (Evens et al., 2017; Supplementary Figure S1).

Plants have evolved two methods of avoiding toxic metals. The first is to exclude metal from the plant, and the second is to enrich the metal elements in a particular organelle. In plants, the roots are responsible for the uptake of metal elements, while the vacuoles are responsible for their exclusion and enrichment. This process involves YSL genes, CDF genes, and ZIP genes (Colangelo and Guerinot, 2006). Aside from transporting zinc and iron, ZIP transporters also transport other metals, including Cd, Ni, and Mn. Most ZIP transporters enhance Zn and Fe at the root when they are expressed, however ZIP gene expression may also enrich Zn and Fe in the stem and leaves (Salt et al., 1995). Fe and Zn are dynamically balanced in plants. Exposing a plant to high concentrations of metal elements destroys the balance between the production and scavenging of free radicals in its cells, which produces a large number of reactive oxygen radicals and induces the peroxidation of unsaturated fatty acids in the membrane. It also causes heavy metal poisoning in plants (Bernstein et al., 2001; Breusegem and Dat, 2006; Zhao, 2007). In this study, we treated three *TaZIP13-B* transgenic lines and one WT line with different concentrations of FeCl_3_ solutions and found that the leaves of the WT developed brown spots and most petioles died. Under ZnSO_4_ treatment, the WT leaves turned gray and the three overexpression lines remained normal. Heavy metals are primarily toxic to plants because they inhibit chlorophyll synthesis, affecting photosynthesis and inducing chlorosis of the leaves (Breusegem and Dat, 2006). *OsIRT*1 overexpression also results in less chlorosis in transgenic plants under Fe-deficient conditions (Lee and An, 2009). In this study, the chlorophyll content in the WT line was significantly lower than in the transgenic lines when exposed to Fe and Zn solutions.

The hormone indole-3-acetic acid (IAA) is related to lateral root formation. Previous studies found that IAA levels increased under Cu and Cd stress but there was no significant change in the roots compared with the control. Zn stress caused significant increases in root branching (Sofo et al., 2013). In this study, the root branch increased under 50μmol/L Zn/Fe stress due to increases in the IAA concentration, resulting in lateral formation. Under MS Zn-/Fe- conditions, the number of lateral roots also increased. Previous studies found that the lateral root of *Arabidopsis* increased under Pi- and Fe-deficient conditions (Rai et al., 2015), while there is no evidence to prove that Zn- or Fe-deficiency promotes the development of lateral roots in *Arabidopsis*. *Arabidopsis* generated different patterns of root system architecture when subjected to different combinations of Pi, nitrate (N), potassium (K), and sulfate (S) deficiencies (Kellermeier et al., 2014). This indicates that *Arabidopsis* has an innate ability to integrate and translate multiple nutrient deficiencies into a complex root developmental program.

Zn and Fe are vital for plant growth and are related to dry matter accumulation in plants, when plants reach the reproductive stage, their photosynthetic products accumulate in the grain. Therefore, the size of the seed is related to the accumulation of dry matter in the early stage of plants (Cock and Yosiida, 1972; Yoshida, 1972; Hirose et al., 2008). However, this increase in seed size is due to overexpression in the plant body, not by seed-restricted expression. This indicates that seed enlargement is due to overexpression in vegetative organs such as the leaves (Hakata et al., 2012). In this study, the seeds of three overexpression lines of *Arabidopsis* were larger than the WT line, indicating that the chlorophyll content in transgenic lines is higher than in WT lines.

We also detected the Fe and Zn content in plant tissues. Previous studies found that the Zn and Fe content in seeds improved when *ZmZIP7*, *ZmZIP3* and *ZmZIP5* and *ZmIRT1* were transferred to wild *Arabidopsis* and maize (Li et al., 2013, 2016, 2019b). Our results demonstrated that the Fe and Zn contents in the roots and shoots were more enriched in overexpression lines than in the WT line and that Fe content was particularly increased in the seeds. This study indicated that *TaZIP13-B* can enrich and transport Fe and Zn in transgenic lines and improve Fe and Zn content in seeds.

## Data Availability Statement

The original contributions presented in the study are included in the article/Supplementary Material, further inquiries can be directed to the corresponding author.

## Author Contributions

WZ and SL conceived and designed the study. SL, ZL, and HL collected the samples. LG, HL, XN, and SC analyzed the data. SL wrote the manuscript. All authors contributed to the article and approved the submitted version.

## Funding

This research was funded by the National Transgenic Key project of Ministry of Agriculture of China (2020ZX08009-15B), Key project of Research and Development Program of Shaanxi (2019NY-014), and the National Natural Science Foundation of China (grant no. 31871611).

## Conflict of Interest

The authors declare that the research was conducted in the absence of any commercial or financial relationships that could be construed as a potential conflict of interest.

## Publisher’s Note

All claims expressed in this article are solely those of the authors and do not necessarily represent those of their affiliated organizations, or those of the publisher, the editors and the reviewers. Any product that may be evaluated in this article, or claim that may be made by its manufacturer, is not guaranteed or endorsed by the publisher.

## Footnotes

^1^ http://plants.ensembl.org/index.html

^2^ http://pfam.xfam.org/

^3^ https://www.ncbi.nlm.nih.gov/

^4^ https://www.evolgenius.info/evolview/#login

^5^ https://wolfpsort.hgc.jp/

^6^ http://gsds.cbi.pku.edu.cn/

^7^ http://meme-suite.org/

^8^ http://www.cytoscape.org/

^9^ http://www.wheat-expression.com/

^10^ https://wheat-urgi.versailles.inra.fr/Tools

^11^ http://www.mirbase.org/

## Supplementary Material

Supplementary Figure S1 Phylogenetic relationship and homoeologs of the 58 TaZIP proteins. The gene that ends with S and L was identified by previous study. Genes highlighted in green were used for yeast complementation. Genes highlighted in yellow were used in this study by yeast complementation.

Supplementary Figure S2 Chromosome locations of these 58 TaZIP genes.

Supplementary Figure S3 Expression patterns of three ZIP genes under Zn- and Fe-deficient conditions. Seedling two leaf of Xiaobaimai shoots (S) and roots (R), under standard nutrient condition (CK), Zn-, Fe-deficiency treated, were harvested, respectively, at 0, 6, and 12h, after treatment. **(A)** Treat with Zn-deficiency; **(B)** treat with Fe-deficiency. Data from real-time RT-PCR experiments were analyzed according to the 2^−∆∆*C*t^ method. Relative mRNA abundance of each gene was normalized with TaActin gene. The error bars indicate standard deviations.
