# Supplementary figures and images for: Genome-Wide Identification of Wheat ZIP Gene Family and Functional Characterization of the TaZIP13-B in Plants
Source: Front Plant Sci. 2021 Nov 3;12:748146. doi: 10.3389/fpls.2021.748146 (PMC8595109; doi:10.3389/fpls.2021.748146)

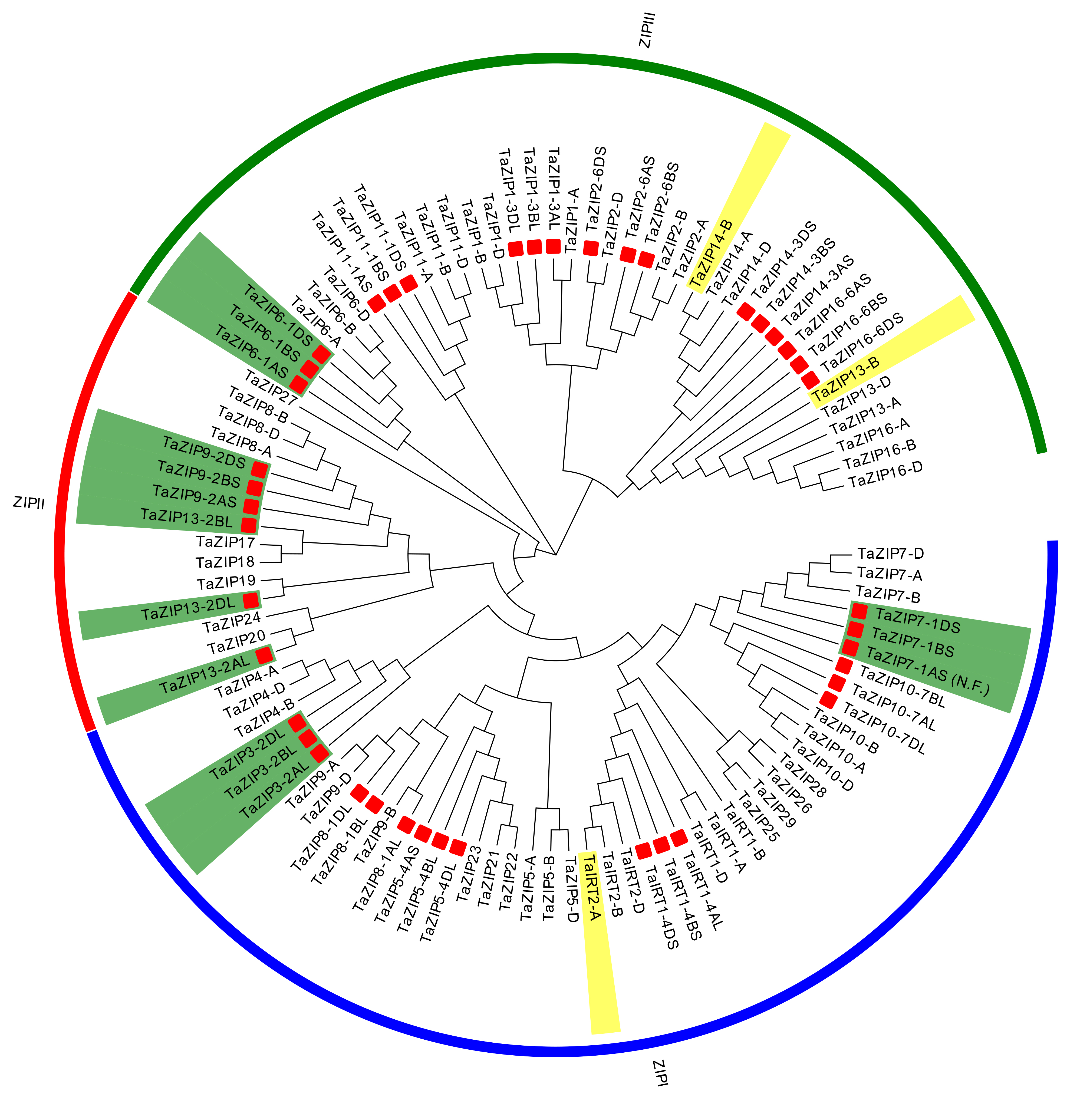

Supplement: Supplementary Figure S1 — Phylogenetic relationship and homoeologs of the 58 TaZIP proteins. The gene that ends with S and L was identified by previous study. Genes highlighted in green were used for yeast complementation. Genes highlighted in yellow were used in this study by yeast complementation. [file Data_Sheet_1.ZIP › Supplementary Figure S1.tif]

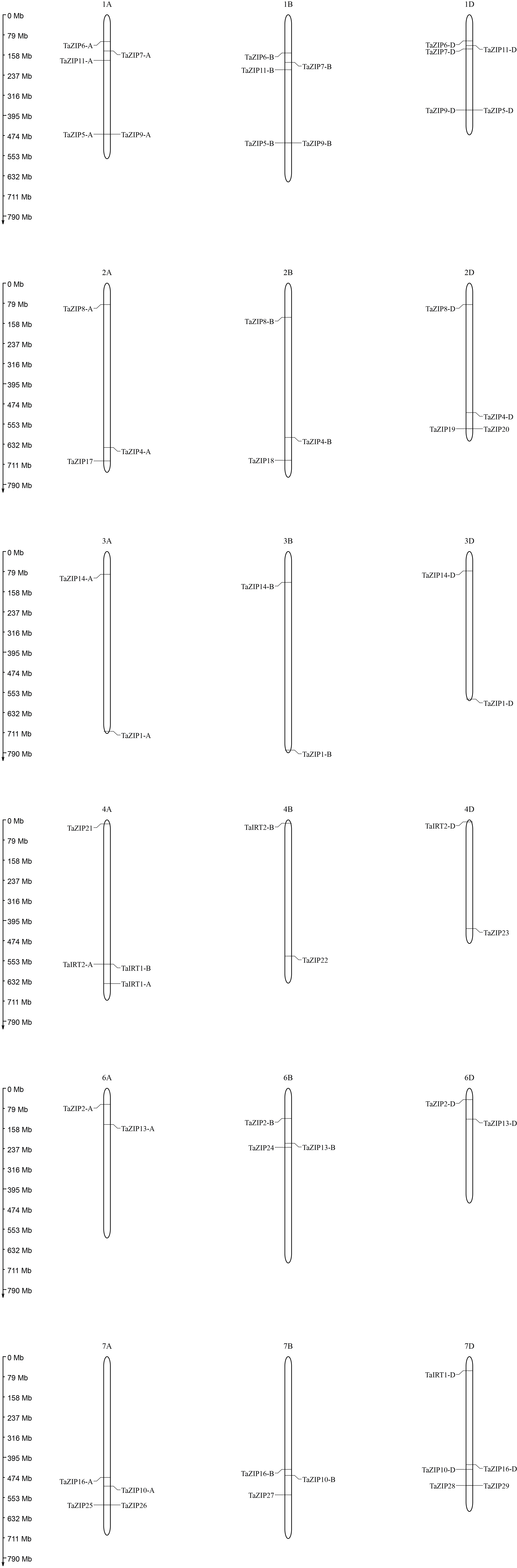

Supplement: Supplementary Figure S1 — Phylogenetic relationship and homoeologs of the 58 TaZIP proteins. The gene that ends with S and L was identified by previous study. Genes highlighted in green were used for yeast complementation. Genes highlighted in yellow were used in this study by yeast complementation. [file Data_Sheet_1.ZIP › Supplementary Figure S2 .tif]

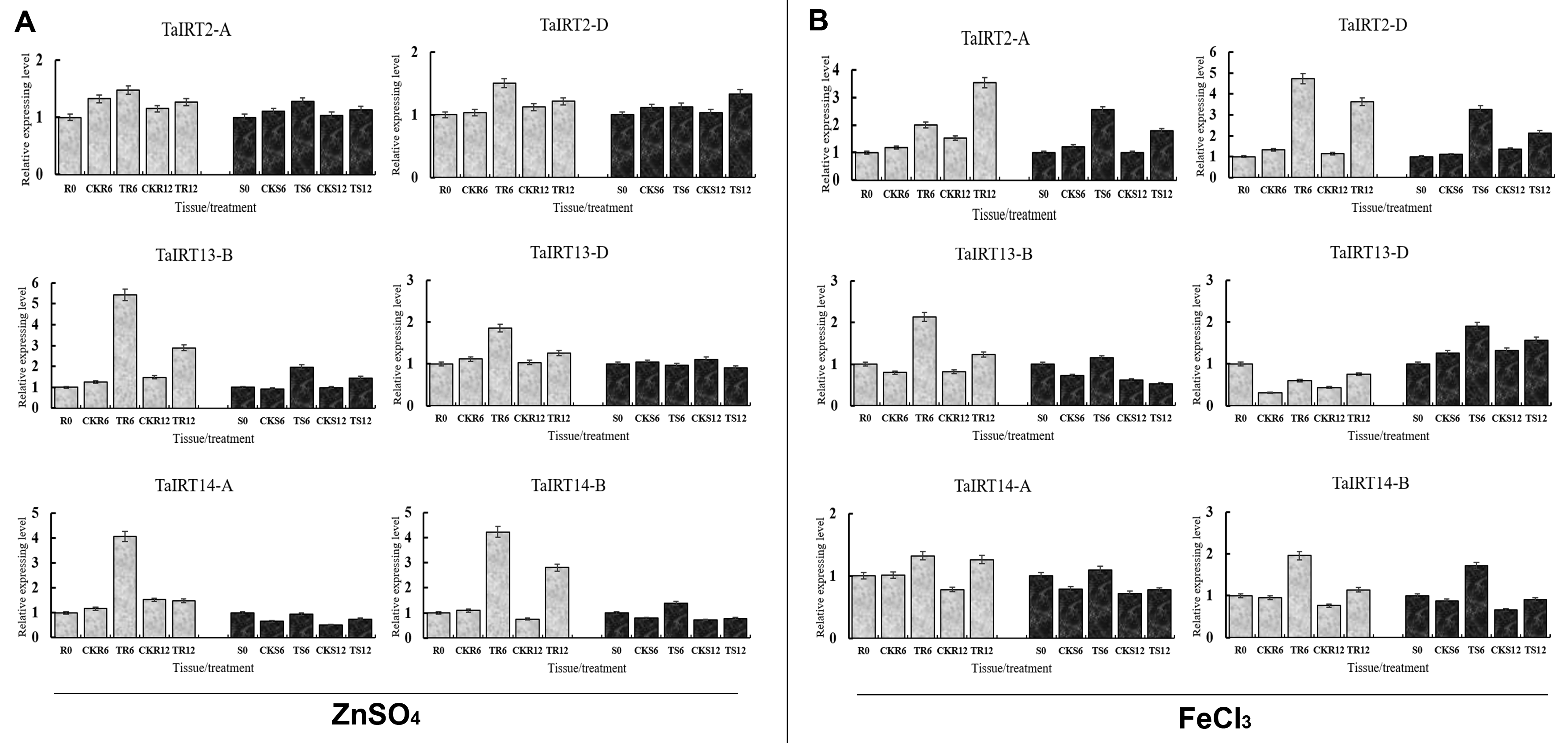

Supplement: Supplementary Figure S1 — Phylogenetic relationship and homoeologs of the 58 TaZIP proteins. The gene that ends with S and L was identified by previous study. Genes highlighted in green were used for yeast complementation. Genes highlighted in yellow were used in this study by yeast complementation. [file Data_Sheet_1.ZIP › Supplementary Figure S3.tif]
